# Targeting pericytes for therapeutic approaches to neurological disorders

**DOI:** 10.1007/s00401-018-1893-0

**Published:** 2018-08-10

**Authors:** Jinping Cheng, Nils Korte, Ross Nortley, Huma Sethi, Yamei Tang, David Attwell

**Affiliations:** 1 Department of Neurology, Sun Yat-Sen Memorial Hospital, Sun Yat-Sen University, 107 Yan Jiang Xi Rd, Guangzhou, 510120 People’s Republic of China; 2 Department of Neuroscience, Physiology and Pharmacology, University College London, Gower Street, London, WC1E 6BT UK; 3 Department of Neurosurgery, National Hospital for Neurology and Neurosurgery, Queen Square, London, WC1N 3BG UK

**Keywords:** Pericyte, Capillary, Blood–brain barrier, Ischaemia, Alzheimer’s, Spinal cord injury, Diabetes

## Abstract

Many central nervous system diseases currently lack effective treatment and are often associated with defects in microvascular function, including a failure to match the energy supplied by the blood to the energy used on neuronal computation, or a breakdown of the blood–brain barrier. Pericytes, an under-studied cell type located on capillaries, are of crucial importance in regulating diverse microvascular functions, such as angiogenesis, the blood–brain barrier, capillary blood flow and the movement of immune cells into the brain. They also form part of the “glial” scar isolating damaged parts of the CNS, and may have stem cell-like properties. Recent studies have suggested that pericytes play a crucial role in neurological diseases, and are thus a therapeutic target in disorders as diverse as stroke, traumatic brain injury, migraine, epilepsy, spinal cord injury, diabetes, Huntington’s disease, Alzheimer’s disease, diabetes, multiple sclerosis, glioma, radiation necrosis and amyotrophic lateral sclerosis. Here we report recent advances in our understanding of pericyte biology and discuss how pericytes could be targeted to develop novel therapeutic approaches to neurological disorders, by increasing blood flow, preserving blood–brain barrier function, regulating immune cell entry to the CNS, and modulating formation of blood vessels in, and the glial scar around, damaged regions.

## Introduction

Capillary pericytes have important roles in blood vessel formation and stabilization [5, 149], blood–brain barrier (BBB) formation and maintenance [6, 11, 29], control of capillary diameter and cerebral blood flow (CBF) regulation [9, 54, 55, 116], amyloid β clearance [124], mediation of neuroinflammation [67, 122, 135], glial scar formation [46], and in some circumstances they exhibit properties of stem cells [33, 98, 109]. Recent studies have revealed that pericytes have important roles in numerous CNS disorders including ischaemic stroke [36, 54, 155], epilepsy [84], spinal cord injury (SCI) [46, 89], diabetes [43], Huntington’s disease [35], Alzheimer’s disease (AD) [55, 124], multiple sclerosis (MS) [122], glioma [20, 53, 138, 148], radiation necrosis [87] and amyotrophic lateral sclerosis (ALS) [150]. In these disorders, pericyte malfunction often leads to BBB disruption and/or a decrease of blood flow, thus causing secondary neurological damage. In this review, we will initially introduce the biological characteristics of pericytes and then discuss how they act either protectively or to promote damage in the progression of CNS disorders. We will focus in particular on the possible mechanisms and consequences for disease of pericyte contraction, pericyte death, abnormal angiogenesis, immunological derangement and scar formation, which will shed light on possible therapies for CNS diseases. We will first consider how pericytes are defined and the heterogeneity of their properties, then review their main physiological functions, before describing how their malfunction contributes to different CNS diseases and suggesting therapeutic approaches that are based on targeting pericytes.

## Defining pericytes and their heterogeneity

**Fig. 1:**
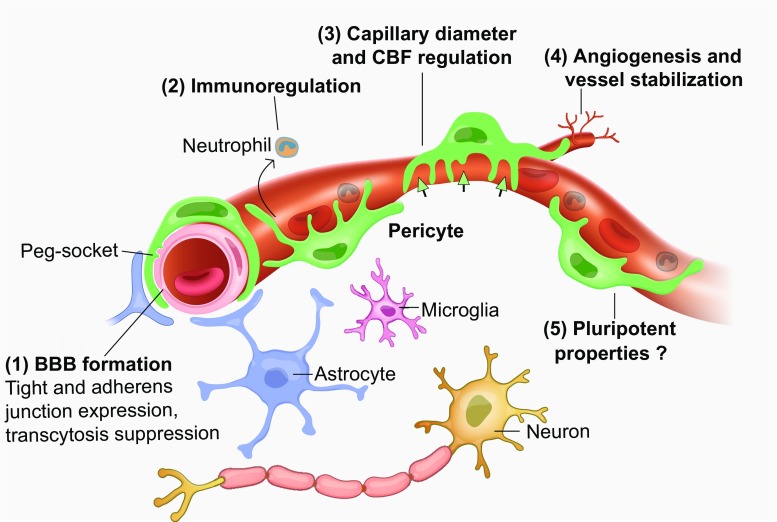
Functions of CNS pericytes in health. Pericytes form a chain contacting endothelial cells [14], interacting physically with them via a peg and socket structure. Several functions of CNS pericytes are illustrated. (1) BBB formation and maintenance, by regulating tight and adherens junctions, and transcytosis across endothelial cells. (2) Immunoregulation by pericytes regulating the entrance and movement of immune cells such as neutrophils. (3) Capillary diameter (arrows) and hence CBF are regulated by α smooth muscle actin-expressing circumferential processes of pericytes on at least the first four branching order vessels of the capillary bed. (4) Angiogenesis and vessel stabilization are mediated by pericytes during the development and repair of the vasculature. (5) Pericytes can proliferate after conditions like ischaemia, and may also be able to differentiate into other cell types

**Fig. 2:**
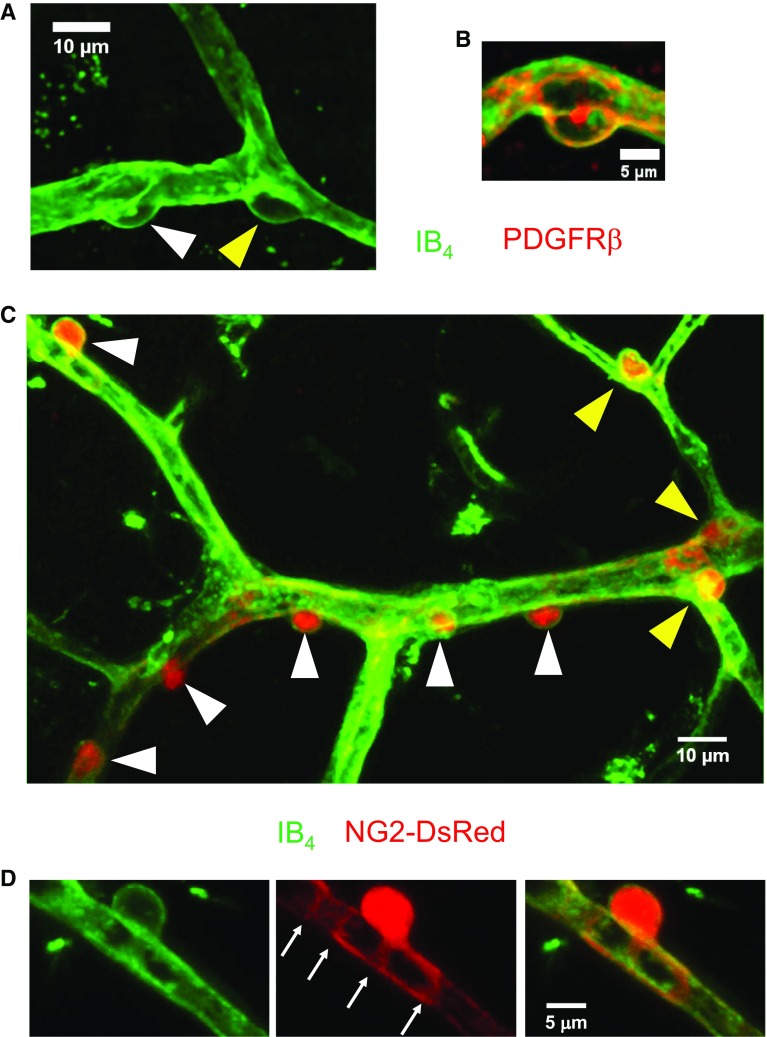
Morphology of, and common labelling methods for, pericytes. **a** Human cortical pericytes, in healthy tissue removed to allow glioma removal. Isolectin B_4_ tagged with a green dye is used to label the basement membrane, which extends along capillaries and around pericytes. Pericytes can be seen on straight parts of the capillary and at junctions [white and yellow arrow-heads, respectively, here and in **c**]. **b** Human cortical pericyte as in **a**, labelled for IB_4_ (green) and the pericyte marker PDGFRβ. **c** Cortical capillaries labelled for IB_4_ (green) in an NG2-DsRed mouse in which pericytes are red. **d** Larger views of the top left pericyte show circumferential DsRed-labelled processes (arrows) that will adjust capillary diameter when they contract

Pericytes, also known as mural cells (a classification which also includes vascular smooth muscle cells around arterioles) or Rouget cells, were first described by the German scientist Ebert in 1871 and the French scientist Rouget in 1873. They were named as “pericytes” by Zimmermann in 1923 [81, 166] due to their location enveloping the endothelium, and being embedded in the basement membrane outside brain vessels including capillaries, post-capillary venules and terminal arterioles (Figs. 1, 2) [5].

Even today, correct identification of pericytes is challenging because of their heterogeneity [5, 49, 59], an understanding of which is likely to be important for understanding their role in disease. For example, there are more contractile pericytes expressing α smooth muscle actin (αSMA) at the arteriole end of the capillary bed [9, 64] (out to the 4th branching order of the capillary bed), there is probably differential regulation of immune cell migration by pericytes at different locations along the capillary bed [135], and a subset of pericytes can proliferate after CNS injury and contribute to the scar which isolates damaged tissue from surrounding healthy tissue [32, 46, 63]. Furthermore, pericytes need to be distinguished from a population of fibroblast-like cells that are present on CNS blood vessels other than capillaries [144].

Nevertheless, platelet-derived growth factor receptor β (PDGFRβ) [90], alanyl aminopeptidase (CD13) [82], the proteoglycan neuron-glial antigen 2 (NG2) [110] and desmin [99] are markers often used to identify pericytes (Fig. 2), with α smooth muscle actin being used to define a contractile sub-class of pericytes [100]. Other markers, such as regulator of G-protein signalling protein 5 (RGS5) [18], SUR2 (ATP-binding cassette transporter subfamily C member 9 or ABCC9) [17], Kir6.1 (potassium inwardly rectifying channel subfamily J member 8) [17], delta-like non-canonical Notch ligand 1 (DLK1) [17], T-box transcription factor 18 (Tbx18) [50], GLAST [46] and endosialin [23], also label pericytes, but in addition label other cells. The expression of all these markers changes during growth and development, and may be up- or downregulated in pathological conditions [5]. Therefore, cell morphology, anatomical position, and the absence of endothelial and glial cell markers, should also be taken into consideration to reduce misidentification of pericytes. In particular, a standard anatomical criterion for defining pericytes on capillaries is that they have spatially isolated nuclei, with processes running along the capillary that separate them from the next pericyte soma along the vessel. This distinguishes them unambiguously from vascular smooth muscle cells (vSMCs), which abut each other when forming the smooth muscle around arterioles. Ignoring this fundamental distinction has led to a recent misidentification [64] of contractile pericytes, which express αSMA and extend processes wrapping around capillaries, as being vSMCs (see discussion in Ref. [9]).

The use of genetic mouse models such as NG2-dsRed [126], NG2-EYFP [160], RGS5-GFP [109], NG2-eGFP [64] and NG2/PDGFRβ-tdTomato [59] mice, which label (at least some classes of) pericytes and their progeny, has paved the way for studying the fate of pericytes in physiology and pathology using intravital imaging. Additionally, the development of NG2- and PDGFRβ-driven Cre expression (constitutive or inducible) which can be crossed with specific floxed mice lines to delete genes of interest, may aid the field in gaining a deeper understanding of the role of pericytes in physiological and pathological settings [25]. Some pericyte-deficient mice, such as Pdgfb^ret/ret^ mice in which the PDGF-B retention motif is depleted to disrupt its binding to heparan sulphate proteoglycans [91] and *Pdgfrβ*^+/−^ or *Pdgf* hypomorph mice, which have a 20–50% reduction of pericytes [6, 11, 29], have also been created to study the effect of pericyte degeneration on neurovascular function. However, the use of some mouse lines requires care. For example, along with being expressed in pericytes, NG2 is also expressed in oligodendrocyte progenitor cells and PDGFRβ is reported to be expressed in some neurons, so understanding the effects of Cre driven by the promoters for these proteins requires control experiments to be sure that the effects seen are mediated by pericytes.

It has recently been reported that a subset of pericytes specifically take up the fluorescent Nissl dye NeuroTrace 500/525 so that they can be distinguished from other brain cells, which may also open a window for studying pericyte behaviour in physical and pathological states [28]. Why this label preferentially enters pericytes is unclear, and it is still uncertain whether this dye labels all pericytes or just the non-contractile pericytes in the middle of the capillary bed.

## Transcriptome studies of pericyte heterogeneity

Ultimately we can hope that transcriptome and proteome studies will define precisely the mRNA and protein expression of pericytes at different positions along the capillary bed, and how they change in disease. However, for “-omics” studies, it is essential to have a marker for the cells to be studied (to isolate and sort the cells), and choosing one particular marker to define pericytes may well lead to the exclusion of certain subclasses of the cells. Indeed, studies to date have given very different results when characterising pericyte mRNA expression.

For example, comparison of five different mural cell (pericyte plus vascular smooth muscle cell) transcriptomes by the Betsholtz group [62] revealed a “surprisingly limited overlap” of the main genes expressed, with the five studies reporting only three “core” transcripts in common. Two of these transcripts were related to actomyosin contraction, yet a subsequent study by the same group [144] reported that pericytes express almost no α smooth muscle actin. This is very surprising, since pericytes are visibly observed to contract and relax in videos provided by several studies [54, 116], and α smooth muscle actin has been observed in immunohistochemical studies of pericytes by numerous groups [2, 9, 10, 64, 156] (even in mid-capillary bed pericytes when actin depolymerisation is inhibited [2]). At present it is unclear whether these discrepancies reflect differences in the method of selecting pericytes for transcriptome analysis, a rapid down-regulation of αSMA expression during processing for the transcriptomic analysis, or mediation of contraction by a previously unappreciated process (perhaps involving *γ* actin [49]).

## Normal functions of pericytes underlying their role in CNS disease

Below we will describe recent data showing how pericytes contribute to CNS disorders. To understand their role in disease, it is essential to understand the normal functions of pericytes and how they may vary between pericytes at different locations on the capillary bed. Here, therefore, we will review the main functions of pericytes, before describing how deficits in these pericyte functions contribute to neuronal damage in disease.

### Blood vessel formation

Pericytes play a key role in the generation of new blood vessels. A complex web of bidirectional signalling pathways mediating interactions between endothelial cells and pericytes is essential for forming new blood vessels and stabilising existing ones (clinical disorders resulting from defects in the operation of these pathways are discussed below). Briefly (for reviews see [134, 139]), endothelial cells release PDGF-BB which binds to PDGFRβ on pericytes, enhancing their proliferation and recruiting them to the endothelial tube. An association of pericytes with capillaries is essential for a capillary to be stable [90], and in the brain it is also a prerequisite for a properly functioning blood–brain barrier (see below). Signalling mediated by pericyte-derived angiopoietin-1 (Ang-1) binding to Tie-2 tyrosine kinase receptors mainly on endothelial cells (but also on pericytes [141]) promotes vessel formation by increasing endothelial cell proliferation, migration and survival [1], while a related ligand, Ang-2 (expressed in developing blood vessels), inhibits the effects of Ang-1. TGF-β1, which is produced by endothelial cells and pericytes, induces the formation of pericytes and inhibits endothelial cell proliferation [5, 13], while in hypoxic conditions VEGF is produced and induces proliferation and migration of pericytes [68, 159]. Together, these pathways, with additional signalling via Notch-3 [146] and NG2, lead to the formation of a fairly regularly spaced array of capillaries (with pericytes located at intervals along them) that deliver adequate oxygen and glucose to the CNS tissue. Interestingly, the ratio of pericytes to endothelial cells is somewhat higher in CNS tissue than in most other vascular beds and varies between CNS locations [5, 38], presumably reflecting some quantitative difference in the strength of signalling by all these pathways that results in an adequate number of pericytes to maintain an adequate vessel density, blood flow and BBB function. Table 1 summarizes the functions and disorders in which these signalling pathways are involved.

**Table 1:** Molecules mediating pericyte–endothelial cell interactions and their associated disorders

Signalling	Function	Dysfunction	Reference
PDGF-BB/PDGFR	Mesenchymal cell differentiation, mural cell proliferation, recruitment, migration, endothelial cell–pericyte attachment	Fahr’s disease (idiopathic basal ganglia calcification, with loss of function mutations in PDGFB and PDGFRB) Ageing and AD (plasma PDGF-BB levels and CSF soluble PDGFRβ levels rise) Amyotrophic lateral sclerosis Diabetes (PDGF-BB level is elevated, hyperglycaemia causes the downstream PDGFRβ signal transduction cascade to induce pericyte apoptosis)	[16, 43, 74, 95, 101, 125, 150]
TGFβ/TGFβR2	Mural cell proliferation, migration, differentiation and survival; promotes expression of contractile and extracellular matrix (ECM) proteins; cooperates with Notch signalling to promote *N*-cadherin expression	Intraventricular haemorrhage Cerebral cavernous malformation Ischaemic stroke	[51, 88, 92, 128, 140, 145]
Ang/Tie2	Maintains the balance of vessel maturation and stability	Diabetes Ischaemic stroke Cerebral cavernous malformation	[26, 162]
Notch	Pericyte survival and expression of *N*-cadherin	Cerebral cavernous malformation Intraventricular haemorrhage Glioblastoma CADASIL (Notch3 mutations)	[44, 69, 80, 106, 128, 146]
VEGF-A/VEGFR2	Promotes cell survival, angiogenesis and vascular permeability	Ischaemic stroke Traumatic brain injury Glioblastoma	[34, 45, 68, 76, 105, 159]

### Constriction and dilation of capillaries

Calcium-dependent contraction of pericyte processes that run around capillaries (Fig. 2d) evokes capillary constriction. This occurs both in response to pericyte depolarization produced by a microelectrode (which is presumed to open voltage-gated Ca^2+^ channels in the pericytes, as will a rise of [K^+^]_*o*_ in pathology), and when a rise in [Ca^2+^]_*i*_ is evoked by a range of neurotransmitters and other vasoactive molecules including noradrenaline, ACh, ATP, angiotensin II, endothelin-1 and lactate [54, 72, 73, 83, 89, 116, 142, 151, 153]. A claim that these constrictions are mediated by vSMCs [64] has been explained to reflect an erroneous definition of pericytes [9]: any contractile cell on vessel walls with circumferential processes was defined in Ref. [64] to be a vSMC, ignoring the conventional (nearly 100 year old) definition of spatially isolated mural cells (even those with circumferential processes) as being pericytes [81, 166]. Noradrenaline release from locus coeruleus neurons confers a contractile tone to pericytes. This allows pericyte relaxation, capillary dilation and an increase in capillary blood flow [15, 54, 79] when neuronal activity releases dilating factors such as glutamate (which evokes ATP release from neurons, thus raising astrocyte [Ca^2+^]_*i*_ and generating prostaglandin E_2_ release), adenosine or lactate, or when NO is released from endothelial cells [27, 42, 54, 116, 152, 153]. When neurons are active, pericytes relax and increase the diameter of capillaries faster than vSMCs relax to dilate penetrating arterioles [54, 79]. Since most of the vascular resistance within the brain parenchyma is located in capillaries [47], and the magnitude of the dilation that pericytes produce of capillaries is similar to that which occurs in penetrating arterioles when neurons are active [54], it follows that pericyte-mediated capillary dilation contributes significantly to the increase of blood flow that is triggered by neuronal activity [15, 54, 79]. Interestingly, even mid-capillary bed pericytes (which mainly lack circumferential processes that could contract to provide tone) may regulate blood flow by adjusting capillary diameter, perhaps by altering growth of the endothelial tube [14] or by relaxing and decreasing vessel wall stiffness when neurons are active to allow easier passive dilation [121].

### Blood–brain barrier maintenance

The BBB is conferred by tight junctions between endothelial cells, and low rates of transcellular vesicular transport (transcytosis) across CNS endothelial cells. Transgenic experiments in which the level of PDGFRβ is reduced, thus reducing the number of pericytes present by up to 50%, have revealed that the presence of pericytes is essential to maintain the BBB [6, 11, 29]. Pericytes achieve this by: (1) increasing endothelial expression of Ang1 and decreasing expression of Ang2, which leads to a suppression of vascular permeability [6]; (2) promoting expression of the major facilitator superfamily domain containing 2a (Mfsd2a) transporter protein in endothelial cells, which in turn suppresses vesicle-mediated transcytosis across the endothelial layer [6, 12, 22, 29]; and (3) maintaining tight junction protein expression in older animals [11]. Loss of pericyte-induced BBB function leads to influx into the brain parenchyma of molecules with a molecular weight up to 500 kDa, including serum proteins (such as thrombin and fibrinogen) and perhaps other toxic molecules (such as glutamate and ATP) that cause neuronal and vascular damage and lead to microglial activation [11]. Pericyte-deficient mice have reduced cerebral blood flow resulting in neurovascular uncoupling, reduced oxygen supply to brain and metabolic stress [79].

### Regulation of immune cell entry

In pathology, immune cells enter the brain. This process is regulated by pericytes. Transgenic deletion of pericytes leads to an upregulation of leukocyte adhesion molecules and plasmalemma vesicle-associated protein (PLVAP) in endothelial cells [29]. PLVAP regulates leukocyte migration both in blood vessels and lymphatic vessels [52, 118]. In muscle it has been shown that leukocytes cross the venule endothelial cell layer and then migrate along pericyte processes before exiting into the tissue at gaps between pericytes [117], while work in the placenta has shown that, having entered the tissue, they are then attracted to capillary pericytes by release of the chemoattractant macrophage migration–inhibitory factor (MIF) [135]. Loss of pericytes leads to leukocytes entering the brain, and may modulate the inflammatory response [29, 122].

### Proliferation and migration in response to injury

CNS injury evokes a recruitment of immune cells to the injury site, but also astrogliosis which leads to the formation of a so-called ‘glial scar’ around the injury site, forming a barrier between the injured and the non-injured tissue which may reduce further neuronal loss, at the possible expense of hindering the regeneration of axons through the lesion [3, 147, 165]. Some cells within the glial scar express NG2, which may contribute to the hindrance of axon regrowth [96]. Although some of these cells are oligodendrocyte precursor cells, a significant fraction of cells within the glial scar are apparently derived by proliferation and migration of a subset of NG2-expressing pericytes that express the glutamate transporter GLAST [32, 46, 63].

## Role in CNS disorders

**Fig. 3:**
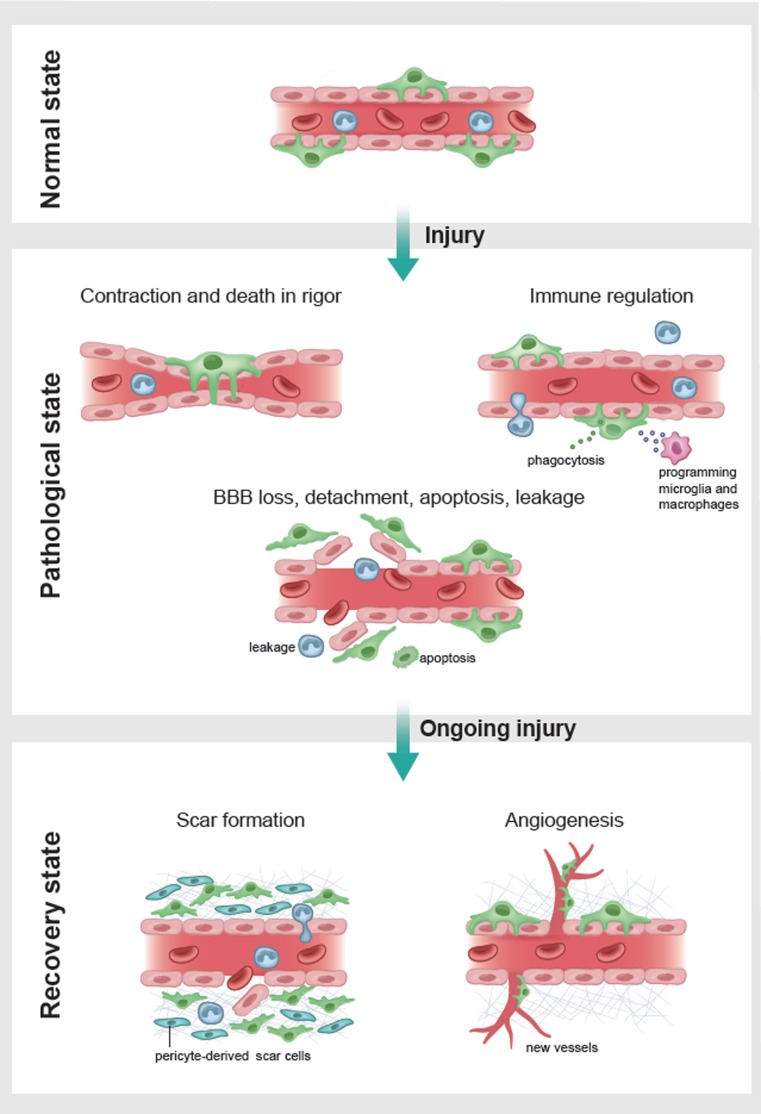
Pericyte responses to brain injury. Injury can: (1) induce pericyte (green) mediated constriction of capillaries; (2) evoke pericyte-mediated regulation of immune function by recruitment of immune cells (leukocytes, blue) to the brain parenchyma, phagocytosis (of green circles) and release of factors (blue open circles) that modulate microglial and macrophage function; and (3) cause loss of BBB function with detachment of pericytes from capillaries and apoptosis, and vessel leakage. Later after injury, pericytes proliferate and migrate to contribute to the scar around the damaged area, and promote blood vessel formation to re-supply the damaged area with nutrients

Given the important roles of pericytes in normal CNS function described above, it is unsurprising that they play a major role in disease. In general, such contributions include alterations in vasculogenesis, capillary diameter, BBB function, immune cell entry and glial scar formation (Fig. 3). In this section, we will review the evidence for these actions in a series of neurological diseases, before considering what drug treatments might be used to target pericytes therapeutically. We deal first with disorders involving energetic challenge and cell depolarization (stroke, the spreading depression occurring in traumatic brain injury and migraine, epilepsy and spinal cord injury), then a situation of energy-oversupply (diabetes), before discussing protein malfunction diseases (Huntington’s and Alzheimer’s), and finally diseases that cannot easily be grouped with others including multiple sclerosis, glioma, radiation necrosis and amyotrophic lateral sclerosis. In general, the evidence cited comes from animal models of disease, but where possible we describe human patient or post-mortem data.

### Stroke

During ischaemia, for example caused by block of an upstream artery, pericytes constrict capillaries both in vitro and in vivo [54, 116, see also 64 but note that this paper mis-named pericytes as smooth muscle cells)]. This is presumably because the fall of ATP levels in pericytes leads to less Ca^2+^ extrusion and a rise of [Ca^2+^]_*i*_—a process facilitated by the large rise of [K^+^]_*o*_ and concomitant depolarization of all cells that occurs during the anoxic depolarization which occurs after a few minutes of ischaemia [57]. In profound ischaemia (“chemical ischaemia”, which prevents synthesis of ATP from either glycolysis or oxidative phosphorylation), this constriction occurs over 15–30 min [54], and a similar pericyte-mediated constriction of coronary capillaries occurs within 45 min when the heart experiences ischaemia in vivo [103]. In the brain this constriction, which will reduce blood flow, is followed by the pericytes dying [36, 54]. This death is thus expected to occur with the pericytes in rigor, constricting the capillaries [54], suggesting that, even after the upstream artery is unblocked by administration of tissue plasminogen activator or use of a stent retriever, a long-lasting decrease of blood flow will occur (until the dead pericytes are removed by microglia). In animal experiments, this so-called no-reflow phenomenon leads to blood flow being reduced by ~ 45% when the upstream artery is unblocked [61], which presumably contributes to continuing generation of neuronal damage. In addition to the loss of blood flow, neuronal damage will also be promoted by any loss of blood–brain barrier function [6, 11, 29] which results from pericyte death.

These data suggest that better restoration of cerebral blood flow and maintenance of BBB function after ischaemia might be achieved by the development of therapies targeted at preventing capillary constriction by pericytes, and preventing pericyte death. Below, we will consider strategies to achieve this. In the longer term after ischaemia, as described below for spinal cord injury, pericytes also proliferate and migrate to contribute to the scar that forms around damaged tissue [36].

### Spreading depression

Cortical spreading depression (SD) is a wave of profound neuronal depolarization triggered either by brain trauma, epilepsy (discussed below) or during migraine attacks. After traumatic brain injury in human patients, the occurrence of SD waves correlates with long-term brain damage [58], probably at least in part due to the reduction of blood flow that occurs. SD is associated with a rise of [K^+^]_o_ to ~ 25 mM, and a prolonged decrease of cerebral blood flow that is generated by release of the vasoconstricting arachidonic acid derivative 20-HETE [37]. Although the involvement of pericytes in the reduction of blood flow remains to be shown, pericyte-mediated capillary constriction by 20-HETE may occur in these conditions, since 20-HETE is known to constrict pericytes [54], and much of the adjustable vascular resistance within the brain parenchyma is located in capillaries [47].

### Epilepsy

In animal models of epilepsy, it has recently been shown that focal capillary constrictions occur in close spatial association (< 3 μm) with NG2-expressing mural cells (pericytes in this case), and that these constrictions are surrounded by regions of neuronal damage [84]. Although the stimulus for the constriction was not studied, a rise of [K^+^]_*o*_ during the seizure might lead to pericyte depolarization and activation of voltage-gated calcium channels, evoking Ca^2+^ entry and pericyte myofilament contraction. Alternatively, the seizure-evoked [K^+^]_*o*_ rise could evoke local release of noradrenaline from locus coeruleus axon terminals, or the seizure-associated rise of [Ca^2+^]_*i*_ might evoke the release of arachidonic acid from astrocytes and generation of vasoconstrictive 20-HETE [8], as occurs in spreading depression [37]. In addition, status epilepticus is associated with increased turnover of pericytes, associated with vessel leakage and a decreased responsivity to glutamate and endothelin [4, 93].

### Spinal cord injury

Spinal cord injury (SCI) often leads to a crushing of blood vessels, generating ischaemia, which may result in pericytes constricting capillaries and dying, as described above. Recently, however, another pericyte-mediated constriction mechanism that decreases spinal blood flow, and produces hypoxia below the lesion, has been revealed [89]. Below the lesion, pericyte 5-HT_1_ and α_2_ adrenergic receptors become activated, evoking capillary constriction, despite the fact that the lesion often leads to a loss of descending monoaminergic neurons. Activation of these receptors results from the production of trace amines (e.g., tryptamine and tyramine) by pericytes that ectopically express the enzyme aromatic l-amino acid decarboxylase (AADC), which synthesizes trace amines from dietary amino acids such as tryptophan [48]. The resulting contraction of pericytes locally constricts capillaries, thus reducing blood flow and causing a chronic state of hypoxia in the spinal cord below the injured site for months in a rat model of SCI. Blocking these amine receptors or AADC was found to restore blood flow and return the tissue oxygen level to normal below the lesion [89], and this improved the motor function and locomotion. Remarkably, inspiring a higher than normal oxygen concentration also raised the oxygen level below the lesion for a prolonged period (~ 20 min), perhaps by increasing neuronal activity and thus evoking the release of vasodilating factors that increased blood flow [89].

Suppression of trace amine generation or blocking the downstream pericyte constriction therefore seem to be promising approaches for partial restoration of function after SCI. The long-lasting effect of raising the local oxygen level by transiently breathing hyperoxic air also suggests the presence of a positive feedback loop, whereby an increase of local blood flow produces a further increase of flow, which might potentiate the effect of therapeutic interventions.

A further aspect of pericyte function after spinal cord injury is that a subset of pericytes (possibly pericytes with properties different from those releasing the trace amines) migrates to form the fibrotic core of the glial scar around the spinal lesion [46]. There is controversy over the functional consequences of the pericyte contribution to the scar, with one study claiming it is needed for revascularisation of the damaged area [63] and another claiming that it reduces axon regrowth through the lesion [32].

### Diabetes

Pericyte loss from retinal capillaries is an early symptom of diabetic retinopathy. The high blood glucose concentration occurring in diabetes activates a pathway, involving protein kinase C δ (PKC-δ) and MAP kinase, which induces a tyrosine phosphatase (SHP-1) to dephosphorylate PDGFRβ and thus inhibit endothelial PDGF-BB signalling to pericytes via this receptor [43]. This leads to pericyte apoptosis, and a decrease in the number of pericytes on capillaries [13, 19]. P2X_7_ receptor activation [137], and increased secretion of Ang2 from endothelial cells acting via the α_3_β_1_ integrin-p53 pathway [56, 114], can also contribute to pericyte apoptosis in diabetes. The loss of capillary pericytes causes loss of blood–retina barrier function. This can be reversed by applying β adrenergic agonists, which may act via Akt [158]. Pericyte loss also leads to microaneurysms [143], which can leak and cause local oedema. Diabetes may similarly induce pericyte loss from brain capillaries [131, 133], although this has been less studied. Pericyte-targeted therapy may thus be useful to protect the retina and brain in diabetes.

### Huntington’s disease

Huntington’s disease (HD) is caused by a mutation (a trinucleotide repeat) in the first exon of the huntingtin gene, which results in a loss of medium spiny neurons in the striatum. HD in humans is also associated with a loss of BBB function, resulting from a decrease in tight junction expression and an increase in endothelial transcytosis, which may reflect a decrease in PDGFRβ expression in pericytes [35]. There is an increase of vessel density, possibly due to more VEGF release from reactive astrocytes, and pericyte density may increase early in the disease but decrease later on [65, 112]. The pericyte changes early in the disease precede neuronal loss [112], and may therefore contribute to that loss.

### Alzheimer’s disease

In Alzheimer’s disease (AD), amyloid β accumulates around the walls of arterioles and capillaries, a condition termed cerebral amyloid angiopathy (CAA). Capillaries in the brains of AD patients also show an abnormal focally constricted morphology [60, 77] with some resemblance to that produced by pericyte contraction [54, 116]. This presumably contributes to the decrease of cerebral blood flow seen in human AD, which is one of the first changes to occur and which can be greater than 40% [7, 66]. This CBF reduction may also, in part, reflect a decrease of the coupling between neuronal activity and blood flow [102], which is partly mediated by pericytes [54, 78, 94]. In human AD and its mouse models, a loss of pericytes from the capillary wall coincides with a loss of blood–brain barrier function [124, 130], consistent with the role of pericytes in maintaining the BBB that was discussed above. Loss of pericytes also appears to promote amyloid β accumulation, tau pathology and early neuronal loss [124]. These changes suggest that therapies aimed at maintenance of normal pericyte function in AD may, by preventing the decrease of CBF and loss of BBB function, serve to preserve neuronal function longer.

### Multiple sclerosis

In multiple sclerosis (MS), peripheral lymphocytes are believed to enter the CNS (a process which may be regulated by pericytes: [29, 52, 117, 118, 135]) and damage myelin and neurons, although there may also be a hypoxic component to the disease [30] which could theoretically induce pericyte loss as in brain ischaemia. Indeed, a loss of BBB function associated with pericyte degeneration is an early feature of human MS [24]. In transgenic mice with low pericyte numbers [31], differentiation of oligodendrocyte precursor cells is slowed during remyelination after a demyelinating insult. Based on culture experiments, this was suggested to reflect laminin 2 derived from pericytes promoting differentiation (and pericytes may also secrete other pro-regenerative molecules [41]), however, interpretation is complicated by the loss of BBB function that occurs in vivo when pericyte number is decreased.

### Glioma

Growing tumours require a supply of energy and carbon skeletons and thus need to become vascularised. In the hypoxic tumour environment, vascular endothelial growth factor (VEGF) is released by the hypoxia and acts on endothelial cells to promote angiogenesis; consequently antibody (bevacizumab) to VEGF has been used clinically to try to suppress blood vessel formation, but patients become resistant to this treatment. Another signalling mechanism that may be worthy of therapeutic attention is the PDGF-BB—PDGFRβ pathway since, to become vascularised, glioma cells express PDGF-BB to attract pericytes to newly formed vessels [53, 138, 148]. Some of the pericytes mediating this function may differentiate from tumour stem cells [20]. In addition, PDGFRβ signalling promotes expression of IL-33 by pericytes, which recruits tumour-associated macrophages that promote tumour metastasis [154]. Therapeutically targeting pericytes to prevent angiogenesis and IL-33 production might thus be used to restrict tumour growth.

### Radiation necrosis

Treatment of tumours with radiation, within or outside the brain, is associated with a loss of pericytes and endothelial cells from the capillaries nearby [87, 127], causing, in the case of the human brain, a leaky BBB and neuronal damage.

### Amyotrophic lateral sclerosis

In human ALS, pericyte loss occurs from spinal cord capillaries, and the magnitude of the loss is correlated with breakdown of the blood–spinal cord barrier and accumulation of blood proteins in the parenchyma [150]. This suggests that prevention of pericyte loss might help to ameliorate the progression of ALS.

## Targeting pericytes to ameliorate brain disorders

Common themes recur in the roles of pericytes in the disorders considered above. In disease, pericytes can:

1. constrict capillaries and reduce cerebral blood flow (epilepsy, stroke, spinal cord injury, and possibly spreading depression and Alzheimer’s disease);
2. cause a loss of BBB function by dying or decreasing PDGFRβ expression (stroke, epilepsy, Huntington’s disease, Alzheimer’s disease, diabetes, multiple sclerosis, radiation necrosis, ALS);
3. migrate to a damaged area to isolate it but may thereby prevent neuronal regeneration through the damage site (spinal cord injury, stroke);
4. be involved in angiogenesis in any insult that involves blood vessel loss or tissue growth (spinal cord injury, traumatic brain injury, stroke, glioma); and
5. probably regulate immune cell entry in the majority of disorders (stroke, spinal cord injury, epilepsy, Alzheimer’s disease, multiple sclerosis).

How can the negative aspects of pericyte function in disease be targeted therapeutically, while promoting the beneficial functions?

### Targeting drugs to CNS pericytes

Two generic issues of developing therapies for CNS pericyte malfunction are: (1) how to get the drug across the BBB, and (2) how to make it act specifically on pericytes.

In some diseases, the BBB will already be defective at sites where pericytes are malfunctioning, perhaps providing an automatic specificity in the brain region where peripherally administered drug action will occur. Alternatively, new methods for achieving penetration of the BBB may be used, such as encapsulating drugs in liposomes or other types of nano-carriers [40, 42, 164].

Some proteins that may be attractive therapeutic targets may happen to be expressed only on pericytes or their interacting endothelial cells (such as Tie2: [161]), while others may be also expressed on other cell types (such as PDGFRβ [161]). For the less selectively expressed targets, it will be necessary to devise drugs that, in addition to recognising their therapeutic target, also bind to another molecule that is selectively expressed on pericytes (or interacting cells).

### Preventing pericyte-mediated constriction of capillaries and pericyte death

Since pericyte-mediated constriction and the pericyte death which occurs in ischaemia are both mediated by Ca^2+^ entry [54, 116], a potential therapeutic approach would be to apply blockers of pericyte voltage-gated Ca^2+^ channels as early as possible after an ischaemic event, for example, when removing an arterial thrombus with a stent retriever or with tissue plasminogen activator. Indeed, voltage-gated Ca^2+^ channel blockers slow ischaemia-evoked capillary constriction in brain slices [104], and also reduce pericyte death evoked by ATP [136] or ischaemia (R. Nortley, F. O’Farrell and D. Attwell, unpublished). Consistent with this, an unpublished study by A. Neuhaus, Y. Couch, B. Sutherland and A. Buchan has found that administering nimodipine at the end of a period of middle cerebral artery occlusion in rats leads to improved blood flow after the simulated stroke and a better behavioural outcome. Similarly, administering nano-carrier-attached adenosine (which may decrease calcium channel activity) maintains capillary dilation after ischaemia and improves behavioural outcome [42]. These approaches may also be beneficial in migraine [75], and after traumatic brain injury or subarachnoid haemorrhage, when pericyte constriction of some capillaries can induce heterogeneity of the transit time for blood flow through the capillaries which decreases oxygen supply to the tissue [107, 108].

The rise of [Ca^2+^]_*i*_ that evokes pericyte constriction of capillaries might be significantly potentiated by pericyte depolarization generated by Ca^2+^-activated chloride channels [151], as in smooth muscle [119]. Block of these channels would offer another possible target for reducing capillary constriction in pathology.

The constriction of pericytes that occurs below spinal cord injuries and leads to local hypoxia, may be relieved by blocking the AADC enzyme that produces trace amines that constrict the pericytes, or blocking the receptors that these amines act on [89]. It is likely that, in other disorders (e.g., spreading depression, epilepsy and Alzheimer’s disease), signalling pathways upstream of pericyte Ca^2+^, or operating in parallel with pericyte Ca^2+^ (such as oxidative stress in ischaemia, which promotes occlusion of vessels by pericytes [156]), will be found that can be blocked to relieve pericyte-mediated capillary constriction.

### Prevention of loss of BBB function

BBB function may be maintained by preventing pericyte death, either as described above for conditions involving a rise of pericyte [Ca^2+^]_*i*_, or by targeting specific death-inducing pathways in other disorders, such as PKC-δ in diabetes [43]. In conditions more mild than those involving pericyte death, BBB function can be improved by promoting interactions between pericytes and endothelial cells, to preserve the activity of Mfsd2a and suppression of transcytosis that are essential for normal BBB function [12, 22]. This can be achieved by increasing PDGF-BB signalling from endothelial cells to PDGFRβ receptors on pericytes (mirroring the loss of BBB function which occurs when PDGFRβ signalling is reduced transgenically [6, 11, 29]), by increasing TGFβ signalling to increase pericyte number, or by modulating Ang2 and Tie2 function [56, 113–115]. Intracerebroventricular administration of exogenous PDGF-BB has entered human clinical trials and appears to be well-tolerated and safe [115]. Thus, in a cell culture model, BBB function is better maintained in hypoxia when PDGF-BB or TGFβ is administered [132]; in status epilepticus, intravenous administration of PDGF-BB reduces blood vessel leakage and normalises blood flow [4]; and in an animal model of Parkinson’s disease, PDGF-BB may restore neurovascular function by rescuing PDGFRβ signalling [111].

Despite its toxic reputation, thalidomide, an immunomodulating agent that is applied in cancer and rheumatic disease [39], can induce pericyte proliferation, recruit pericytes to capillaries, and thus induce vessel maturation, mainly by increasing PDGF-BB expression in endothelial cells [86]. An increased density of pericytes on capillaries would be expected to promote the integrity of the BBB and indeed, in an animal model of AD, administering thalidomide decreases BBB leakiness [123]. Thalidomide has been successfully used to treat hereditary haemorrhagic telangiectasia in humans [86], and has been patented for use to prevent loss of BBB function after radiation therapy [85]. A beneficial effect of thalidomide on pericyte survival has also been confirmed in sunitinib-induced cardiotoxicity (caused by a decrease of PDGFRβ signalling) [21] and radiation-induced kidney injury [127].

Another therapeutic approach to maintaining BBB function is to apply the inhibitor of phosphodiesterase type 3, cilostazol, or the prostacyclin analogue iloprost which activates Gs-coupled IP receptors, both of which are expected to raise the level of cyclic AMP in pericytes. These agents have been shown to reduce the detachment of pericytes and astrocyte endfeet from endothelial cells in stroke-prone rats [105], to preserve BBB function in white matter subjected to demyelination with lysophosphatidylcholine [97], and (in a cell culture model of the BBB) to preserve BBB function in the face of oxygen–glucose deprivation by upregulating tight junctions between endothelial cells [140], apparently by inhibiting TGFβ signalling. This suggests further therapeutic approaches could be targeted to TGFβ signalling.

The importance of PDGFRβ signalling for maintaining pericyte number and BBB function has been exploited to provide a biomarker for AD progression. Loss of pericytes and degradation of BBB function has been shown to correlate with human cognitive decline and the appearance of soluble PDGFRβ in the cerebrospinal fluid [95]. In the long term, it will highly desirable to develop similar assays for other aspects of pericyte function, including TGFβ and Ang-Tie signalling, and ideally to extend such an approach to allow simple blood tests to be used to assess CNS pericyte function.

### Control of immune cell entry in pathology

Numerous neurological conditions are associated with recruitment of immune cells from the blood to the brain. Whether modulating this entry by targeting pericyte functions (such as MIF release [135]) could provide beneficial therapies will depend on whether the net effect of immune cell recruitment is damaging or positive (e.g., releasing anti-inflammatory factors that suppress deleterious microglial actions [129]).

### Controlling pericyte migration into the glial scar

At present, little is known about the factors stimulating pericytes to proliferate and move to damaged areas to contribute to the glial scar. However, blocking proliferation can lessen the pericyte contribution to the scar, which may promote axon regrowth [32] or alternatively hinder revascularisation [63]. Periostin expressed by pericytes is a key molecule involved in regulating pericyte movement into the scar [157], and genetic or pharmacological inhibition of its function decreases pericyte proliferation and scar formation, and improves long-term outcome after spinal cord injury [157].

### Regulating glioma growth

Pericytes contribute to tumour growth both by promoting angiogenesis and by releasing IL-33 to promote metastasis (see above). Both of these actions are driven by PDGF-BB—PDGFRβ signalling, implying that tumour growth may be limited by agents blocking this signalling, such as imatinib and sunitinib (although these drugs also block other tyrosine kinases) [120].

Pericytes may also be a useful target in facilitating access of drugs to tumours, by disrupting the blood–tumour barrier, while leaving the blood–brain barrier less affected. By inhibiting the tyrosine kinase BMX found in stem cell-derived pericytes [20] with ibrutinib, it has recently been shown [163] that chemotherapy agents can have better access to the brain tumours.

### Pericytes as stem cells

A growing body of evidence indicates that pericytes can become multipotential stem cells [33]. Pericytes have been suggested to acquire the ability to differentiate into neuronal, microglial and vascular lineage cells after brain pathology, in conditions such as ischaemic diseases and hypoxia [70, 71, 98, 109]. Thus, reprogramming of pericytes might be employed to promote neurogenesis and vasculogenesis at sites of brain injury. However, all these studies employed ex vivo culture of pericytes to reprogramme their fate, and the idea of pericytes or vascular smooth muscle becoming stem cells has been challenged by a study [50] showing that they do not intrinsically exhibit differentiation potential in vivo during ageing or in pathology.

## Conclusions

Despite being relatively neglected components of the CNS, the data reviewed in this article demonstrate that pericytes, located at the interface between CNS cells and the blood supply coming from the periphery, play numerous crucial roles in the healthy CNS. As a result, they offer many opportunities for therapeutic intervention in a broad range of neurological disorders. We predict the widespread development of pericyte-targeted therapies in the next 10 years.
